# Platelet Adhesion and Aggregation Dynamics over Collagen- and VWF-coated Surfaces: Insights from Dissipative Particle Dynamics Simulations and Microfluidic Experiments

**DOI:** 10.1007/s11538-026-01615-5

**Published:** 2026-03-04

**Authors:** Anik Tarafder, Shigang Wang, Yilin Wu, Dong Han, Bartley P. Griffith, Zhongjun J. Wu

**Affiliations:** 1 Department of Surgery, University of Maryland School of Medicine, 10 South Pine Street, MSTF 436, Baltimore, MD 21201 USA; 2 Fischell Department of Bioengineering, A. James Clark School of Engineering, University of Maryland, College Park, Maryland, USA

**Keywords:** Dissipative particle dynamics, Platelet adhesion, Thrombosis, Hemostasis, Clot formation, Microfluidic experiment

## Abstract

Platelet adhesion and aggregation at sites of vascular injury play a critical role in both physiological hemostasis and pathological thrombosis. Platelets rely on their surface receptors to form bonds with von Willebrand factor (VWF) and exposed subendothelial collagen to initiate the clotting process. Clot formation is a complex, multistep phenomenon involving mechanical and biochemical signaling that activates key surface receptors and drives morphological changes to form aggregates. Here, we perform dissipative particle dynamics (DPD) simulations in combination with experiments using collagen- and VWF-coated microfluidic channels perfused with human blood to investigate platelet adhesion and aggregation mechanisms under physiological flow conditions. We use a viscoelastic spring-dashpot system to model platelet-platelet and platelet-coated surface interactions and introduce a probabilistic approach to capture platelet attachment or detachment on collagen- and VWF-coated surfaces. We quantitatively compare our numerical simulation with experimental results and explain the observed dynamics. Our simulation results show scattered multilayer clots over collagen-coated surfaces, and single-layer, isolated platelet adhesion on VWF-coated surfaces, corroborating experimental observations. The platelet-covered area over collagen-coated surfaces monotonically increases over time, whereas it reaches a plateau after a period of perfusion on VWF-coated surfaces.

**Supplementary Information:**

The online version contains supplementary material available at 10.1007/s11538-026-01615-5.

## Introduction

Despite technical advances in mechanically assisted circulation (MAC), such as extracorporeal membrane oxygenation (ECMO) and ventricular assist device (VAD) therapies, patients frequently experience both bleeding and thrombotic complications (Miller and Rogers 2018; Pinney et al. 2017; Hastings et al. 2017, Oliver, 2009). Shear-induced platelet receptor shedding and abnormal platelet activation under high shear stress have been identified as major contributing factors (Chen et al. 2020b). Hemostasis is an essential physiological process that prevents excessive blood loss by forming a stable clot at sites of vascular injury. When a blood vessel is injured, the normal hemostatic response involves complex interactions among the vascular wall, platelets, coagulation proteins, and shear forces within the bloodstream (Andrews and Berndt 1998; Packham 1994; Wang et al. 2012; Weitz and Eikelboom 2016, Wu and Thiagarajan, 1996). Unregulated or excessive activation of this process can lead to thrombosis, in which a blood clot (thrombus) forms within a vessel obstructing blood flow or embolizes, potentially causing severe complications such as stroke (Tosenberger et al. 2012; Wang et al. 2020).

Platelets play a central role in both physiological hemostasis and pathological thrombosis. In response to vascular injury or prothrombotic conditions such as hypercoagulation or elevated intracellular calcium, platelets adhere to exposed subendothelial matrix and aggregate to form a clot (Barnes et al. 1998; Yip et al. 2005). Platelet surface receptors, such as the glycoprotein (GP) Ibα, GPVI, GPIIb/IIIa, and $\alpha_{2} \beta_{1}$ integrin, mediate platelet interactions by binding to von-Willebrand factor (VWF), collagen, fibrinogen, and other adhesive ligands during these processes (Manon‐Jensen et al. 2016; Wang et al. 2012). Platelet adhesion to the damaged vessel wall begins when the GPIbα binds to surface-immobilized VWF, which acts as a molecular bridge between platelets and exposed components of the extracellular matrix (Moroi et al. 1996). This initial binding is relatively weak and primarily functions to decelerate circulating platelets near the injury site (Patel et al. 2003). Stable adhesion is reinforced by interactions involving GPVI and $\alpha_{2} \beta_{1}$ integrin with collagen, and further by activated GPIIb/IIIa binding to fibrinogen to promote platelet-platelet aggregation into a multilayered firm clot (Patel et al. 2003). Without functional initial GPIbα-VWF and GPVI-collagen interactions, late GPIIb/IIIa activation, and additional platelet recruitment, platelets may adhere as scattered clusters or single layers but fail to form larger, stable multilayered clots (Chen et al. 2020b).

Both computational and experimental investigations have advanced our understanding of platelet adhesion and clot formation dynamics under physiological and pathological conditions (Li et al. 2025; Watson et al. 2024; Gao et al. 2024; Chen and Ruggeri 2024; Keneally et al. 2023; Han et al. 2022; Kaneva et al. 2021; Wang et al. 2020; Tosenberger et al. 2012). More recently, coarse-grained molecular dynamics simulations have provided valuable insights into the role of platelet receptor-ligand interactions in clot formation (Li et al. 2025; Kaneva et al. 2021; Gupta et al. 2019; Zhang et al. 2014; Kamada et al. 2010). Kamada et al. (Kamada et al. 2010) performed coarse-grained molecular dynamics simulations to model platelet adhesion on coated-surface using an elastic spring model, while Tossenberg et al. (Tosenberger et al. 2012) investigated clot collapse mechanisms using time-dependent and step-function elastic models. Kaneva et al. (Kaneva et al. 2021) performed detailed dissipative particle dynamics (DPD) simulations to investigate the dynamic structure of the clot surface (thrombus shell) during formation using a stochastic adhesion model. More recently, Li et al. (Li et al. 2025) performed coarse-grained simulations to understand the effect of VWF unfolding on platelet adhesion using a stochastic approach to model bond formation and breaking between platelet-VWF particles.

Most previous computational approaches only focused on platelet activation, adhesion, and aggregation at vascular injury sites where incoming platelets attach to initiate the clot formation process (Kaneva et al. 2021; Wang et al. 2020; Tosenberger et al. 2016, 2012). However, none of these models considered the scattered, heterogeneous platelet adhesion and aggregation patterns observed experimentally over collagen- and VWF-coated surfaces (Chen et al. 2020b). In vitro studies show that platelets form distinct single-layer adhesion on to VWF-coated surface but develop multilayered big clots over collagen-coated surfaces (Chen et al. 2020a). Previous numerical efforts to understand platelet aggregation over collagen- and VWF-coated surfaces relied largely on advanced simulation techniques that could qualitatively predict the dynamics; however, their results lacked quantitative comparison with experimental results (Li et al. 2025; Kaneva et al. 2021; Tosenberger et al. 2016, 2012; Wang et al. 2020). Tosenberger et al. (Tosenberger et al. 2016, 2012) performed two-dimensional (2D) DPD simulations using a Hookean spring force to model platelet adhesion, clot formation, and rupture mechanism under flow. Their primary focus was to illustrate clot core formation due to the fibrin mesh and did not examine binding to collagen or VWF specifically. Additionally, the deterministic spring forces used to represent adhesive interactions did not consider flow-induced formation and breakage of the key bonds between surface receptors and ligands, which is essential to address the dynamic nature of the thrombus shell (Patel et al. 2003). While Kaneva et al. (Kaneva et al. 2021) proposed a stochastic coarse-grained platelet-adhesion model taking into account the dynamic nature of the thrombus shell and GPIb-mediated bond formation and breaking with VWF-coated surface, due to the 2D simulations, their results could not fully reproduce the heterogeneous, scattered platelet adhesion over VWF-coated surfaces-let alone collagen-coated surfaces-observed during experiments (Chen et al. 2020b). Li et al. (Li et al. 2025) performed three-dimensional (3D) DPD simulations to investigate VWF stretching to mediate platelet adhesion but did not include a stochastic bond formation and breaking mechanism in their simulation.

Here, we developed a 3D coarse-grained DPD model, and performed simulations to investigate clot formation dynamics over collagen- and VWF-coated surfaces. We used the viscoelastic model proposed by Wang et al. (Wang et al. 2020) to model platelet adhesion, which includes fibrin-enhanced clot core formation. We have introduced a stochastic approach to model scattered clot formation dynamics observed during microfluidic channel experiments, where the channel bottom wall is coated with collagen or VWF. The 3D simulations provide accurate capture of the observed collagen- and VWF-specific platelet adhesion dynamics. The viscoelastic model provides both attractive and dissipative phenomena through its spring-dashpot system, and the stochastic adhesion model introduces rapid bond formation and breakage during thrombus formation. We compare our numerical simulations with in vitro experiments to validate our model and explain the observed dynamics.

### Mathematical Modeling and Numerical Method

The platelet adhesion and aggregation over collagen- and VWF- coated surfaces are modeled using the DPD approach, which is based on the coarse-grained molecular dynamics, where each particle represents a cluster of molecules instead of an individual molecule (Espanol and Warren 1995; Groot and Warren 1997; Liu et al. 2015). These particles move according to Newton’s second law of motion and interact with each other with varying forces according to their interparticle distance. The details of the mathematical modeling approach can be found in our previously published article (Wang et al. 2020), and here we briefly present the mathematical equations governing the problem. The particle motion and position are governed by conservative ${\mathbf{F}}_{ij}^{{\mathrm{C}}}$, dissipative $\left( {{\mathbf{F}}_{ij}^{{\mathrm{D}}} } \right)$, and random $\left( {{\mathbf{F}}_{ij}^{{\mathrm{R}}} } \right)$ force acting between two particles *i* and *j*, and calculated as,

1 $$ \frac{{d{\mathbf{r}}_{i} }}{dt} = {\mathbf{v}}_{i} $$

2 $$ \frac{{{\mathrm{d}}{\mathbf{v}}_{i} }}{{{\mathrm{dt}}}} = \frac{1}{{m_{i} }}\mathop \sum \limits_{i \ne j} \left( {{\mathbf{F}}_{ij}^{{\mathrm{C}}} + {\mathbf{F}}_{ij}^{{\mathrm{D}}} + {\mathbf{F}}_{ij}^{{\mathrm{R}}} } \right) $$

3 $$ {\mathbf{F}}_{ij}^{{\mathrm{C}}} = \omega_{c} \left( {r_{ij} } \right){\mathbf{e}}_{ij} $$

4 $$ {\mathbf{F}}_{ij}^{{\mathrm{D}}} = - \gamma \omega_{D} \left( {r_{ij} } \right)\left( {{\mathbf{e}}_{ij} .{\mathbf{v}}_{ij} } \right){\mathbf{e}}_{ij} $$

5 $$ {\mathbf{F}}_{ij}^{{\mathrm{R}}} = \varphi \omega_{R} \left( {r_{ij} } \right)\frac{{\theta_{ij} }}{{\sqrt {dt} }}{\mathbf{e}}_{ij} $$

Here, ${\mathbf{r}}_{i} ,{\mathbf{v}}_{i}$, and $m_{i}$ represent the position, velocity, and mass of particle *i*, while ${\mathbf{r}}_{ij} = {\mathbf{r}}_{i} - {\mathbf{r}}_{J}$, and ${\mathbf{v}}_{ij} = {\mathbf{v}}_{i} - {\mathbf{v}}_{j}$ represent the displacement and relative velocity vector between particles *i* and *j*. ${\boldsymbol{e}}_{ij} = \frac{{{\mathbf{r}}_{ij} }}{{\left| {r_{ij} } \right|}}$ is the unit vector. $\varphi = \sqrt {2\gamma k_{B} T}$ is the random force coefficient, where $\gamma$ measures the strength of dissipative force, $k_{B}$ is the Boltzmann constant, *T* is the temperature. $\theta_{ij} = \theta_{ji}$ is a normally distributed random variable with zero mean and unit variance. The ratio between cut-off radius $r_{c}$ and the distance $r_{ij}$ determines the strength of the three weight functions $\omega_{c} , \omega_{D}$, and $\omega_{R}$, which are represented as,

6 $$ \omega_{c} \left( {r_{ij} } \right) = \left\{ {\begin{array}{*{20}l} a_{ij} \left( {1 - \frac{{r_{ij} }}{{r_{c} }}} \right);&\quad  r_{ij} \le r_{c}  \\ 0 ; &\quad  r_{ij} \ge r_{c}  \\ \end{array} } \right. $$

7 $$ \omega_{R} \left( {r_{ij} } \right) = \left\{ {\begin{array}{*{20}l} \left( {1 - \frac{{r_{ij} }}{{r_{c} }}} \right);& \quad r_{ij} \le r_{c}  \\ 0 ;& \quad r_{ij} \ge r_{c}  \\ \end{array} } \right. $$

8 $$ \omega_{D} \left( {r_{ij} } \right) = \left( {\omega_{R} \left( {r_{ij} } \right)} \right)^{2} $$

where, $a_{ij}$ measures the strength of the conservative force.

### Platelet Adhesion on Collagen and VWF-Coated Surface

Previously, we simulated platelet adhesion and aggregation onto collagen and VWF using a viscoelastic model (Wang et al. 2020). Here, we extend our previous work to model distinct scattered clot formation dynamics over collagen- and VWF-coated surfaces observed in vitro in microfluidic channels (see sec. 3 for in vitro setup). In consistent with experimental settings, the bottom surface of the microfluidic channel is fully covered with collagen- or VWF-tagged particles, and a stochastic approach (de Rooij and Kuhl 2018) is adopted to model scattered clot formation dynamics. The viscoelastic model, along with the stochastic attachment/detachment approach, is implemented to capture the platelet adhesion and aggregation dynamics over the coated surfaces.

The viscoelastic adhesion model is a spring-dashpot system (Tosenberger et al. 2012; Wang et al. 2020), where the elastic and viscous forces determine the strength of the platelet-platelet or platelet-coated surface bonds. The viscoelastic model equations are,

9 $$ {\mathbf{F}}_{ij}^{E} = a_{p} \left( {1 - \frac{{r_{ij} }}{{r_{c} }}} \right){\mathbf{e}}_{ij} , a_{p} = \left\{ {\begin{array}{*{20}c} { - a_{0} ;r_{c} \ge r_{ij} > r_{a} } \\ {a_{1} ;r_{ij} \le r_{a} } \\ \end{array} } \right. $$

10 $$ {\mathbf{F}}_{ij}^{V} = - \gamma_{p} \left( {1 - \frac{{r_{ij} }}{{r_{c} }}} \right)^{2} \left( {{\mathbf{e}}_{ij} \cdot {\mathbf{v}}_{ij} } \right){\mathbf{e}}_{ij} , r_{ij} \le r_{c} $$

Here, $a_{0}$ and $\gamma_{p}$ determine the final clot size, and $a_{1}$ is only present to prevent particle overlapping. $r_{a}$ is set as $r_{a} = \frac{{2r_{c} }}{3}$ and does not significantly affect clot size. A constant value of $a_{1} = 1200$ is used for all simulations to reduce model complexity. Equations (9) and (10) are specific implementations of the platelet-platelet and platelet-coated surface bonds, with a linear spring law and a velocity‐dependent damping term. These equations ensure that platelets undergo a pulling force and a viscous drag within the cutoff distance $r_{c}$ during bond formation or break-up, consistent with the physical phenomena of receptor–ligand binding (Mori et al. 2008).

The conservative spring (elastic) term ${\mathbf{F}}_{ij}^{E}$ acts as a linear Hookean spring that binds approaching platelets to adhered platelets or coated surface particles within a cutoff distance $r_{c}$ (with stiffness $a_{0}$), and switches to a strong repulsion at very small separations (via $a_{1}$) to avoid overlap. The dashpot (viscous damping) term ${\mathbf{F}}_{ij}^{V}$ opposes relative motion along the bond with a magnitude proportional to velocity (scaled by $\left( {1 - \frac{{r_{ij} }}{{r_{c} }}} \right)^{2}$). Thus, the total bond force has both conservative (elastic) and dissipative (viscous) components. $a_{0}$ influences how easily platelets are pulled together, and $\gamma_{p}$ determines how quickly energy is dissipated. These two parameters therefore control the balance of adhesion versus dissipation in the forming clot and thus influence the resulting clot size and structure.

To model the single platelet adhesion and the formation of platelet aggregates (cluster of adhered platelets) over the coated surfaces, we use a probability-based attachment/detachment approach. The probability *P* of a platelet detaching from either an adhered platelet or a coated surface particle, due to a constant external fluid force *F***,** within a time interval $\Delta t$, is given by (de Rooij and Kuhl 2018),

11 $$ P_{dis} \left( {F, \Delta t} \right) = 1 - e^{{\left( { - k\left( F \right)\Delta t} \right)}} $$

12 $$ k\left( F \right) = \left\{ {\begin{array}{*{20}l} k_{0} ;& \quad  F \ll F_{0}  \\ k_{0} e^{{\frac{F}{{F_{0} }}}} ;&\quad otherwise \\ \end{array} } \right. $$

$k\left( F \right)$ represents force-dependent bond detachment rate, where $k_{0}$ determines zero-force constant detachment rate, and $F_{0}$. acts as a characteristic adhesion force scale, similar to the Bell’s model (Bell 1978) for capturing receptor-ligand rupture under force. This approach has been used previously to model the similar receptor-ligand bond formation and breaking in many biophysical systems (Li et al. 2025; Kaneva et al. 2021; de Rooij and Kuhl 2018; Chen and Springer 2001). Equation (11) along with Eq. (12) ensures that platelet-platelet or platelet-coated surface bonds are more stable under low external fluid force and break up rapidly at higher values. At high shear rates, the probability $P_{dis}$ becomes very large, indicating rapid bond rupture, whereas under low shear rates $P_{dis}$ remains small (bonds rarely breaks). These two regimes are captured through the implementation of Eq. (12). The detachment of platelet from another adhered platelet or protein-coated surface depends on $k\left( F \right)$ which switches from base-rate $k_{0}$ at low forces to an exponentially enhanced rate at high forces. Thus, the stability of a platelet-platelet-coated-surface bond decays exponentially with $k\left( F \right){\Delta }t$. In the limit of $F \ll F_{0}$, the bond is strong enough, and external force ${\mathbf{F}}$ has negligible effect on the detachment probability.

### Simulation Setup

**Fig. 1:**
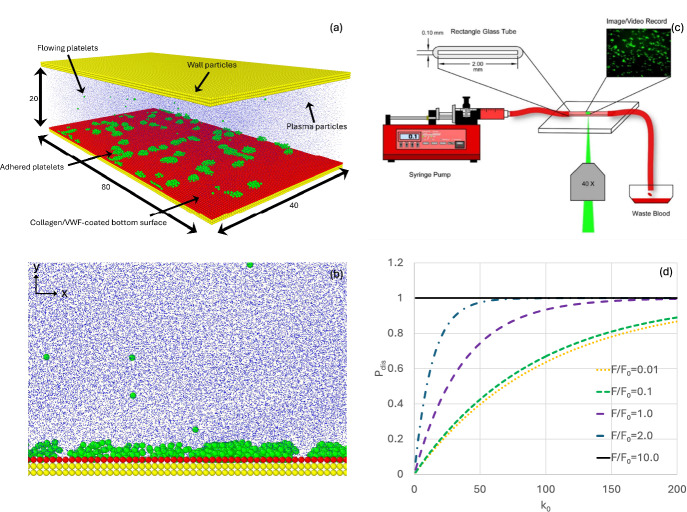
Schematic of the computational and experimental setup. **a** Shows the computational domain dimensions (DPD scale), along with four types of particles used in the numerical DPD simulation: platelet particles (green), collagen/VWF (red), plasma (blue), and yellow (wall particles). Plasma particle diameters are scaled down 10 times for better visualization. **b** Side view of a portion of the computational domain showing platelet adhesion. **c** Shows the experimental setup for investigating platelet adhesion. **d** Shows variation in detachment probability $P_{dis}$ for different model values of eqs. (11 and 12) (Color figure online)

The numerical simulations were performed using the open-source software package LAMMPS developed and distributed by Sandia National Laboratories (Plimpton et al. 2007). Simulation parameters were chosen to closely match the in vitro conditions to accurately analyze scattered clot formation patterns on the collagen- and VWF-coated microchannels. All variables were non-dimensionalized based on the cut-off distance $l_{DPD} = 5\mu m$, unit energy $E_{DPD} = 4.14 \times 10^{ - 21} \frac{{kgm^{2} }}{{s^{2} }}$, unit time $t_{DPD} = 15.86 ms$, and unit mass $m_{DPD} = 4.17 \times 10^{ - 14}$ kg values (Wang et al. 2020). The DPD units were calculated as follows, $E_{DPD} = K_{B} T$, $m_{DPD}$=$\rho l_{DPD}^{3} /n_{s}$, $t_{DPD} = r_{c} \left( {\frac{{m_{DPD} }}{{E_{DPD} }}} \right)^{1/2}$, and $F_{DPD} = K_{B} T/l_{DPD}$. The computational domain is a rectangular channel with dimensions of $80 \times 20 \times 40 l_{DPD}^{3}$ (equivalent to $400 \times 100 \times 200 \mu m^{3}$), where only walls are present in the *y* direction, and bottom wall particles are tagged to represent either the collagen- or VWF-coated surface (Fig. 1a). Platelets, $3\mu m$ (Noris et al. 2014) in diameter, are randomly introduced at the channel entrance and collected at the outlet (evaporation) (Wang et al. 2020). Plasma particles are present in the computational domain and interact with platelet and wall particles with DPD forces. Wall particles are stationary with a number density of 7.5, and plasma particles are mobile with a number density of 3 (Groot and Warren 1997; Zhang et al. 2014). Figure 1a shows a schematic of the computational setup, and Fig. 1b shows a portion of the channel in the *xy* plane.

In experiment (see details in the following section), a 0.1 *ml/min* flow through the microchannel (50 × 0.1 × 2 mm) generates a maximum velocity of ~ 12.5 mm/s at the channel center ($3U_{avg} /2$), and a shear rate of ~ 500 s^−1^ near channel walls, calculated as $\frac{{4U_{max} }}{H}$ (White, 2015) where $H = 0.1 mm$ is the channel height. To be consistent with the experimental values, a constant body force was applied to each DPD particle to achieve a shear-rate close to the experimental value, and a value of $F_{DPD} = 0.45$ was chosen which generated a maximum velocity 11.22 mm/s (see Fig. S1a of Supplement). A polynomial curve was fitted to generate a continuous velocity profile (see Fig. S1b of Supplement), and the derivative of the fitted equation provided the shear-rate value for the DPD simulation, which is calculated as ~ 517 s^−1^. The corresponding Reynolds number, $Re = \rho U_{avg} L/\mu$ ~ 0.04, for $\rho = 1000kg/m^{3}$, and $L = 5 \mu m$, indicating a viscous-dominated flow. Note that the velocity profile plot (see Fig. S1a of the Supplement) shows significant slip velocity near the wall, due to the nature of the coarse-grained simulation, and was previously addressed in the literature (Pivkin and Karniadakis 2005). In DPD simulations, fluid particles tend to penetrate wall particles when the particle number density and conservative force coefficients of the fluid and wall particles are identical. Increasing the wall particle number density or the conservative force coefficient, in combination with a “bounce-back” boundary condition between wall and fluid particles, has been shown to mitigate wall penetration (Pivkin and Karniadakis 2005). Similarly, here, we have used a higher particle number density (7.5), and a “reflect” wall-fluid particle interactions. Although this approach is simple and straightforward, it often introduces slip velocity at the wall (Pivkin and Karniadakis 2005). Improved accuracy may be achieved by applying a special treatment to the conservative force coefficients, calculated as an effective wall-fluid conservative force coefficient (Pivkin and Karniadakis 2005). We did not apply any special treatment to the wall-fluid interaction, which might have introduced slip velocity near the wall observed in Fig. S1b of the Supplement. As the shear-rate of DPD is close to the experimental value, the overall hydrodynamic effect on the platelet adhesion is expected to be sufficiently equivalent. All model parameters and their physical values are listed in Table 1. As outlined in our previous work (Wang et al. 2020), various simulations were run with different sets of viscoelastic parameters $a_{0}$ and $\gamma_{p}$ until the platelet covered area percentage matched with the experimental observations. Briefly, viscoelastic parameter $a_{p}$ was kept fixed, and $\gamma_{p}$ was varied until a spatial distribution of the adhered platelets was achieved, $a_{p}$ was then varied to get a stable adhesion. During each set of parameter runs, multiple simulations with varying $k_{0}$ and $F_{0}$ were performed to match final experimentally measured platelet adhesion levels.

**Table 1:** Model parameters and their corresponding physical values

Parameters	Model value	Physical value
Length, ${\boldsymbol{l}}_{{{\boldsymbol{DPD}}}}$	1	5 μm
Solvent density, ${\boldsymbol{\rho}}$	1	1000 kg/m^3^
Fluid particle number density, ${\boldsymbol{n}}_{{\boldsymbol{s}}}$	3	
Mass, ${\boldsymbol{m}}_{{{\boldsymbol{DPD}}}}$=$\user2{\rho l}_{{{\boldsymbol{DPD}}}}^{3} /{\boldsymbol{n}}_{{\boldsymbol{s}}}$	1	$4.17 \times 10^{ - 14}$ kg
Cut-off radius, ${\boldsymbol{r}}_{{\boldsymbol{c}}}$ Platelet diameter	1 0.6	5 μm 3 μm
Energy, ${\boldsymbol{E}}_{{{\boldsymbol{DPD}}}} = {\boldsymbol{k}}_{{\boldsymbol{B}}} {\boldsymbol{T}}$	1	4.14 × 10^−21^ kg m^2^/s^2^
Time, ${\boldsymbol{t}}_{{{\boldsymbol{DPD}}}} = {\boldsymbol{r}}_{{\boldsymbol{c}}} \left( {\frac{{{\boldsymbol{m}}_{{{\boldsymbol{DPD}}}} }}{{{\boldsymbol{E}}_{{{\boldsymbol{DPD}}}} }}} \right)^{1/2}$	1	15.86 ms
Velocity, ${\boldsymbol{V}}_{{{\boldsymbol{DPD}}}}$	1	0.32 mm/s
Wall particle number density, ${\boldsymbol{n}}_{{\boldsymbol{w}}}$	7.5	
Kinematic viscosity of plasma, ${\boldsymbol{\nu}}$	1	1 × 10^−6^ m^2^/s
Boltzmann constant, ${\boldsymbol{k}}_{{\boldsymbol{B}}}$	1	1.38 × 10^−23^ J/K
Temperature, *T*	1	300 K
Conservative force coefficient, ${\boldsymbol{a}}_{{{\boldsymbol{ij}}}} = 75{\boldsymbol{k}}_{{\boldsymbol{B}}} {\boldsymbol{T}}/{\boldsymbol{n}}_{{\boldsymbol{s}}} {\boldsymbol{r}}_{{\boldsymbol{c}}}$	25 (plasma-wall) 10 (plasma-plasma, plasma-platelet plasma-coated surface)	2.07 × 10^−14^ N 8.28 × 10^−16^ N
Dissipative force coefficient, ${\boldsymbol{\gamma}}$	4.5 (plasma-wall, plasma-plasma, plasma-platelet)	3.73 × 10^−15^ N
Viscoelastic conservative force coefficient (collagen-coated surface), ${\boldsymbol{a}}_{0}$	50 (platelet-platelet) 400 (platelet-collagen)	4.14 × 10^−14^ N 3.31 × 10^−13^ N
Viscoelastic dissipative force coefficient (collagen-coated surface), ${\boldsymbol{\gamma}}_{{\boldsymbol{p}}}$	4.5 (platelet-platelet) 80 (platelet-collagen)	3.73 × 10^−15^ N 6.62 × 10^−14^ N
Viscoelastic conservative force coefficient (VWF-coated surface), ${\boldsymbol{a}}_{0}$	150 (platelet-platelet) 400 (platelet-VWF)	1.24. × 10^−13^ N 3.31 × 10^−13^ N
Viscoelastic dissipative force coefficient (VWF-coated surface), ${\boldsymbol{\gamma}}_{{\boldsymbol{p}}}$	80 (platelet-platelet) 80 (platelet-VWF)	6.62 × 10^−14^ N 6.62 × 10^−14^ N
Random force coefficient, $\phi = \sqrt {2\user2{\gamma k}_{{\boldsymbol{B}}} {\boldsymbol{T}}}$	3	2.48 × 10^−15^ N
Bond detachment rate (collagen-coated surface), ${\boldsymbol{k}}_{0}$	120 (platelet-platelet) 120 (platelet-collagen)	7566 1/s
Bond detachment rate (VWF-coated surface), ${\boldsymbol{k}}_{0}$	10 (platelet-platelet) 80 (platelet-VWF)	630 1/s 5044 1/s
Characteristic bond force, ${\boldsymbol{F}}_{0} = 1000{\boldsymbol{k}}_{{\boldsymbol{B}}} {\boldsymbol{T}}/{\boldsymbol{r}}_{{\boldsymbol{c}}}$	1000 (platelet-platelet, platelet-coated-surface)	8.28 × 10^−13^ N
Repulsive force coefficient, ${\boldsymbol{a}}_{1}$	1200	9.94 × 10^−13^ N
Timestep, ${{\boldsymbol{\Delta}}}{\boldsymbol{t}}$	0.01	0.1586 ms

Platelet-plasma, plasma-plasma, and inactivated platelet-platelet particle interactions are governed by the DPD flow equations as described in eqs. (1)–(4). Platelet adhesion is modeled in a two-stage fashion to mimic the physiological process, where stage one represents reversible GPVI- or GPIb $\alpha$-induced adhesion ($t_{DPD}$ = 0.01), and stage two ($t_{DPD} = 300$) mimics irreversible fibrin enhanced clot formation through GPIIb/IIIa receptor (Wang et al. 2020). Adhered and activated platelets recruit incoming inactivated platelets, activate them, and form fibrin-enhanced firm bonds (Wang et al. 2020). $k_{0}$ and $F/F_{0}$ together determine detachment probability $P_{dis}$ in each timestep, according to eqs. (11) and (12), and Fig. 1d shows the variation of $P_{dis}$ with $k_{0}$ for various values of $F/F_{0}$. For each platelet-platelet or platelet-collagen interaction, $F/F_{0}$ varies locally due to the fluid force *F*, and, at a certain value of $k_{0}$, a range of $P_{dis}$ can be expected for each interaction. For example, at a rate of $k_{0} = 100$, a $P_{dis}$ between 0.62 and 1 can be expected depending on local force ratio $F/F_{0}$. To model scattered clot formation (Fig. 1a) over the collagen- and VWF-coated surfaces, at each timestep $\Delta t$, we generate a random viable $P_{r}$. . If $P_{r} \ge P_{dis}$. , the incoming platelet attaches to an adhered platelet or to collagen- or VWF-coated surface particles. If $P_{r} < P_{dis}$, the platelet detaches from the connected platelets or from collagen or VWF-coated surfaces. Thus, at each timestep ${\Delta }t$, platelets can attach to or detach from adhered platelets or coated-surface particles, which introduces the stochastic nature of platelet adhesion. The timestep ${\Delta }t = 0.01$ (physical time ~ 0.16 ms) was selected for all simulations and reducing ${\Delta }t$ to a smaller value (${\Delta }t = 0.005$) does not significantly change the platelet covered area percentage over time (see Fig. S2 of the Supplement).

Equations (1) and (2) are integrated based on the Verlet method (Martys and Mountain 1999) to find the positions and velocities of the particles. The simulations were performed for ~ 5 min of physical time to match the experimental duration. The platelet-covered area percentage and the number of adhered platelets over the coated surfaces are the primary focus of this study. The platelet-covered area was divided by the total area of the bottom surface to calculate the covered area percentage, which was then compared with experimental results.

## Experimental Method

Fresh human blood was collected from healthy adult donors by venipuncture into a sterile blood collection bag containing sodium citrate (3.8%; 0.109 mM). All donors had not taken antiplatelet or anticoagulant medications for two weeks before blood donation. Informed consent from the donors was obtained in accordance with the Declaration of Helsinki and the study was approved by the Institutional Review Board of the University of Maryland Baltimore.

The schematic of the experimental setup for platelet adhesion on collagen- or VWF-coated surface is shown in Fig. 1c. Rectangular glass capillary tubes (VitroTubes 50 × 0.1 × 2 mm, VitroCom, Mountain Lakes, NJ, USA) were coated with collagen (1 mg/mL, Chrono‐log, Havertown, PA, USA) or VWF (100 µg/mL, EMD Millipore) and incubated overnight at 4°C in a humidified box. The tubes were then blocked with 1% bovine serum albumin (BSA) in phosphate-buffered saline (PBS) at room temperature for 1 h and rinsed with PBS. Platelets in whole blood were labeled with mepacrine (20 μM) (Sigma, St. Louis, MO, USA) for 20 min. A fluorescent microscope (IX71, Olympus, Tokyo, Japan) with an Olympus DP80 digital camera was used to visualize platelet adhesion dynamics. Blood samples were perfused through the coated glass tubes for 5 min in the dark using a syringe pump (NE-300, New Era Pump Systems Inc., Farmingdale, NY, USA) at a controlled flow rate (~ 0.1 mL/min, shear rate of 500 s^–1^). Digital videos were recorded at 1 frame/s on a personal computer using Olympus CellSens software. For platelet adhesion on VWF and collagen-coated surfaces, a custom MATLAB (Natick, MA, USA) program calculated the area covered by adhered platelets in each frame over time. The Fiji TrackMate framework was used to track the rolling motion of adhered single platelets (Ershov et al. 2022).

## Results

**Fig. 2:**
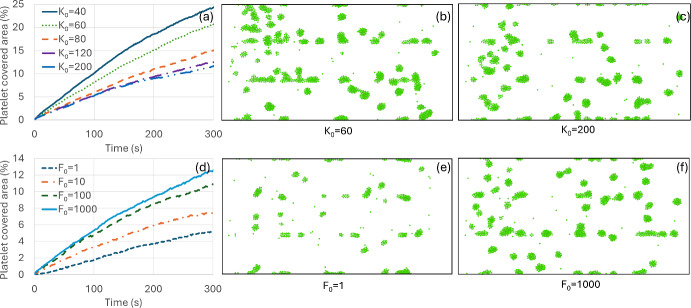
Variation in platelet-covered area percentage for various probabilistic model values of $k_{0}$ and $F_{0}$. **a** Shows variation in platelet-covered area with time for various values of $k_{0}$. (**b**) and (**c**) show platelet-covered area snapshots from DPD simulation at t = 300s for $k_{0}$=60 and 200. **d** Shows variation in platelet-covered area with time for various values of $F_{0}$. (**e**) and (**f**) shows platelet-covered area snapshots from DPD simulation at t = 300s for $F_{0}$=1 and 1000. Note that plasma, coated surface, and wall particles are removed for visualization

Figure 2 shows variations in the platelet-covered area percentage with time for various values of $k_{0}$ and $F_{0}$ along with their two representative snapshots at time $t = 300s$. It shows the influence of the stochastic adhesion model parameters on the platelet-covered area for general platelet-coated surface particle interactions (not specific to collagen or VWF). Figure 2a shows variations of $k_{0}$ while keeping the other model parameters fixed at $a_{0} = 80$, $\gamma_{p} = 4.5$, $F_{0} = 1000$(platelet-platelet), and $a_{0} = 400$, $\gamma_{p} = 80$, $F_{0} = 1000$ (platelet-coated surface). Figure 2b and c show platelet-covered area snapshots at time 300 s for $k_{0} = 60$ and $200$. With the increase of $k_{0}$ from 40 to 200, the platelet-covered area percentage decreases from ~ 24% to ~ 12% (Fig. 2a, b, and c) at t = 300 s. This is expected as with the increase in detachment rate $k_{0}$ from 40 to 200 increases the detachment probability $P_{dis}$ almost twice (Fig. 1d). Increasing $F_{0}$ from 1 to 1000 increases the cover area percentage more than twofold, indicating strong influence of the initial bond force and the force ratio ($F/F_{0}$) on the covered area (Fig. 2d, e, and f), as was also observed in Fig. 1d.

**Fig. 3:**
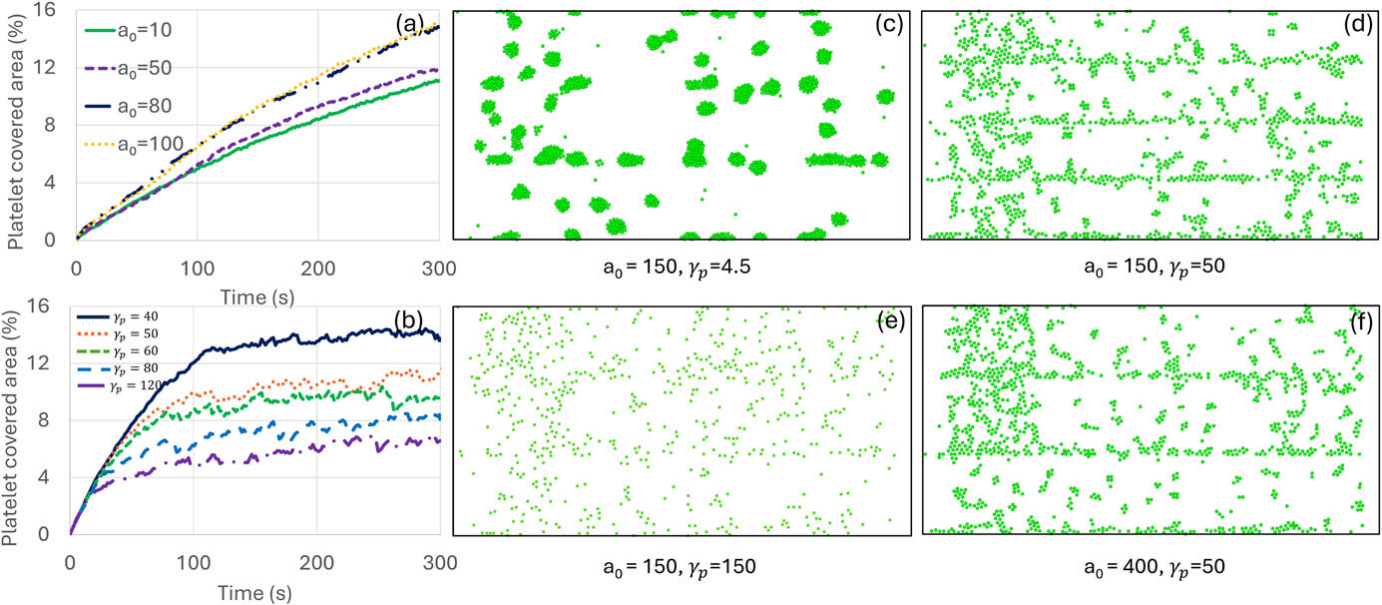
Variation in platelet-covered area percentage for various viscoelastic model values of $a_{0}$ and $\gamma$. **a** and **b** show variation in platelet-covered areas with time for various values of $a_{0}$ and $\gamma_{p}$. (**c**), (**d**), (**e**), and (**f**) show platelet-covered area snapshots from DPD simulation at t = 300s for various values of $a_{0}$ and $\gamma_{p}$. Note that plasma, coated surface, and wall particles are removed for visualization

Figure 3 shows variations in clot formation pattern over a coated surface with time due to the change in viscoelastic model parameters. It represents the variations in platelet adhesion dynamics (single platelet adhesion over VWF-coated surfaces and platelet aggregates formation over collagen-coated surfaces) due to the change in the elastic parameter $a_{0}$ and the dissipative parameter $\gamma_{p}$. These two parameters help to simulate the experimentally observed platelet aggregate formation phenomena over collagen-coated surfaces, and single platelet adhesion over VWF-coated surfaces. As same viscoelastic model acts between platelet-platelet and platelet-coated surface particle interactions, we fix the parameters $a_{0} = 400$, and $\gamma_{p} = 80$ for platelet-coated surface particle interactions for all our simulations and vary platelet-platelet interaction parameters ($a_{0}$ and $\gamma_{p}$) to capture distinct platelet adhesion dynamics over collagen- and VWF coated surfaces. Figure 3a shows the change in platelet-covered area percentage for various values of $a_{0}$ for platelet-platelet interactions over time keeping $\gamma_{p} = 4.5$ the same for all. At a specific time, with the increase in elastic coefficient $a_{0}$, the platelet-covered area increases (Fig. 3a). Figure 3b shows the variation in platelet-covered area for different values of dissipative coefficient $\gamma_{p}$ for the platelet-platelet interactions, keeping elastic coefficient fixed at $a_{0}$= 50. The platelet-covered area decreases with the increase in $\gamma_{p}$, indicating less adhesion. Figure c, d, e, and f show the snapshots of platelet aggregates for various sets of model parameters. An increase in $\gamma_{p}$ introduces single isolated-type platelet adhesion (Fig. 3d, e, and f) as opposed to platelet aggregates (Fig. 3c). An approximately ten times increase in $\gamma_{p}$ leads to distinct patterns of clots on the coated surface: multilayered clustered type (Fig. 3c) for $\gamma_{p} = 4.5$, and isolated single platelet adhesion (Fig. 3d, e and f) for $\gamma_{p} > 40$. In contrast, for the same value of $\gamma_{p}$, an increase in $a_{0}$ slightly increases the number of adhered platelets (Fig. 3d and f). The increase in damping effect ($\gamma_{p}$) reduces relative motion (as explained in Sec. 2.2) between an already adhered platelet and an incoming platelet, thus, rather than attaching on top of the adhered platelet, the incoming platelet slides pass it and attaches over a nearby VWF-coated surface. The stochastic model introduces (eqs. (11) and (12)) force-dependent platelet adhesion and detachment from the coated surface, whereas the viscoelastic model (via $a_{0}$ and $\gamma_{p}$ of eqs. (9) and (10)) determines distinct platelet adhesion patterns (platelet aggregates over the collagen-coated surface (Fig. 3c), and single platelet adhesion over the VWF-coated surface (Fig. 3d, e, and f).

**Fig. 4:**
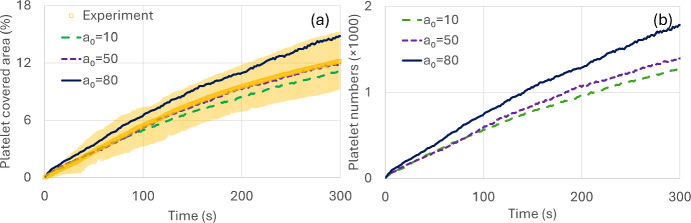
Comparison between DPD simulation-predicted and experimentally observed platelet adhesion over the collagen-coated surface. **a** Shows DPD simulated platelet-covered area percentage variation with time along with experimental results. The shaded region indicates the span of the 8 experimental data, and square markers represent their average value. **b** Total platelet numbers in the clots

**Fig. 5:**
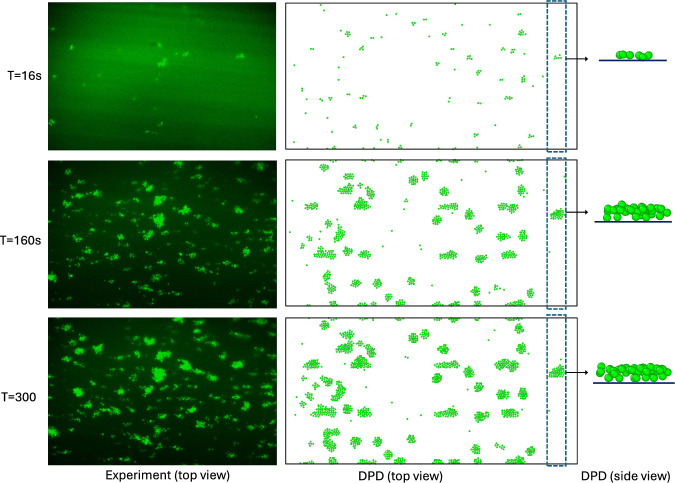
Snapshot images of platelet-covered areas over the collagen-coated surfaces at three different times from experiments (left), and DPD simulation (right). The experiment shows only the top view, and DPD shows both the top view and side view of a portion of the covered area. Note that plasma, coated surface, and wall particles are removed for visualization

Figure 4a presents simulated and experimental platelet-covered area percentage varying with time over the collagen-coated surface. Numerical results were plotted for three values of $a_{0}$, where the platelet-covered area percentage increases with time. Experimental data are obtained from eight experimental observations, where the symbols show the average value and the colored shaded region shows the span of the data. Numerical results match experimental data quite accurately and capture the clot formation trend observed over time. Figure 4b shows total platelet numbers in the clots which increase monotonically with time, following the increasing trend in the platelet-covered area percentage over time. Figure 5 shows snapshots of DPD-simulated (right) and experimentally observed (left) platelet aggregation on the collagen-coated surface at three time points (t = 16s, 160s, and 300s) for a 5 min simulation. Simulated data were obtained with the model parameters $a_{0} = 50$, $\gamma_{p} = 4.5$, $k_{0} = 120$ which were adjusted to match the in vitro experimental condition (Fig. 4a) closely. In vitro observations show scattered clot formations on the coated surface with brighter colors representing larger multilayered clots. The DPD simulation quite accurately predicted the experimentally observed trend.

**Fig. 6:**
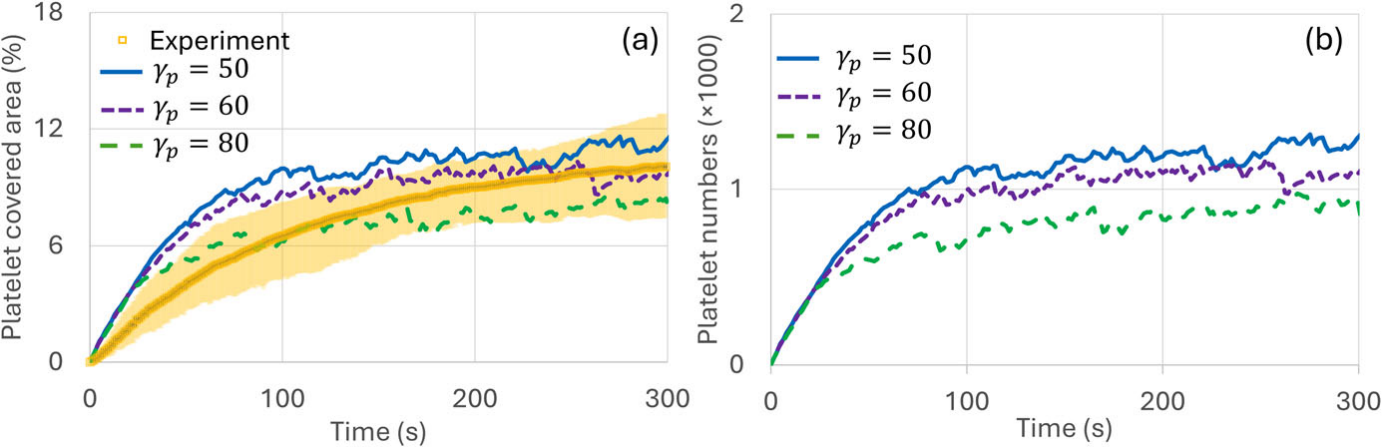
Comparison between DPD simulation predicted and experimental platelet adhesion over the VWF-coated surface. **a** Shows DPD simulated platelet-covered area percentage variation with time along with experimental results. The shaded region indicates the span of the 8 experimental data, and square markers represent their average value. **b** Total platelet numbers in the clots

**Fig. 7:**
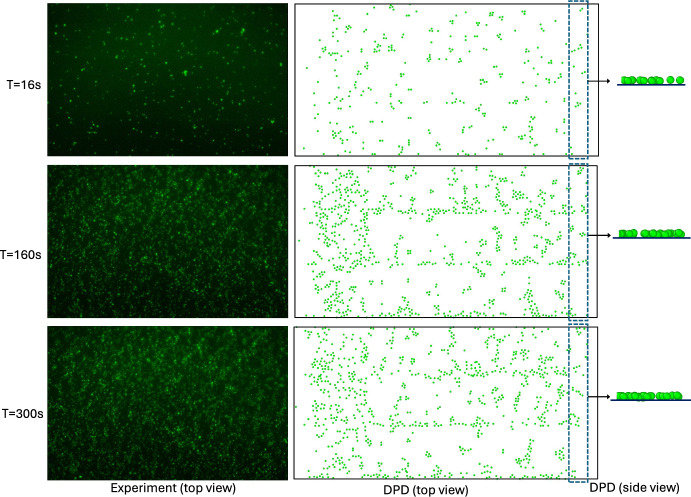
Snapshot images of the platelet-covered areas over VWF-coated surfaces at three different times from experiments (left), and DPD simulation (right). The experiment shows only the top view, and DPD shows both the top view and side view of a portion of the covered area. Note that, plasma, coated surface, and wall particles are removed for visualization

Figure 6a presents simulated and experimental platelet-covered area percentage varying with time over the VWF-coated surface. Numerical results are plotted for three values of $\gamma_{p}$ along with the experimental observations. As described before in Fig. 3, increasing $\gamma_{p}$ helps to capture single platelet adhesion dynamics (Fig. 3d, e, and f), rather than platelet aggregates (Fig. 3c) typically observed over the VWF-coated surfaces. Thus, we fix $a_{0}$ and vary $\gamma_{p}$ to simulate platelet adhesion over VWF-coated surface. Experimental data are obtained from eight experimental observations, where the symbols show the average value, and the colored shaded region shows the span of the data. Numerical results matched well with experimental data and captured the platelet adhesion trend observed over time. Figure 6b shows total platelet numbers in the clots which increase monotonically with time, following the increasing trend in the platelet-covered area percentage. Platelet-covered areas and total adhered platelet numbers increased with time up to 100 s and the rate (slope) slowed down after ~ 100 s indicating a saturation. Single platelet adhesion on the VWF-coated surface has been captured quite accurately and matched with experimental observations. Figure 7 shows simulated (right) and experimental (left) platelet-covered area over the VWF-coated surface at three different times t = 16s, 160s, and 300s for a 5 min simulation. Simulated data were obtained with the model parameters $a_{0} = 50$, $\gamma_{p} = 80$, $k_{0} = 120$. In vitro observations show single isolated-type platelet adhesion on the VWF-coated surface. The DPD simulation predicted the experimentally observed trend showing scattered, single isolated-type platelet adhesion on the VWF-coated surface.

**Fig. 8:**
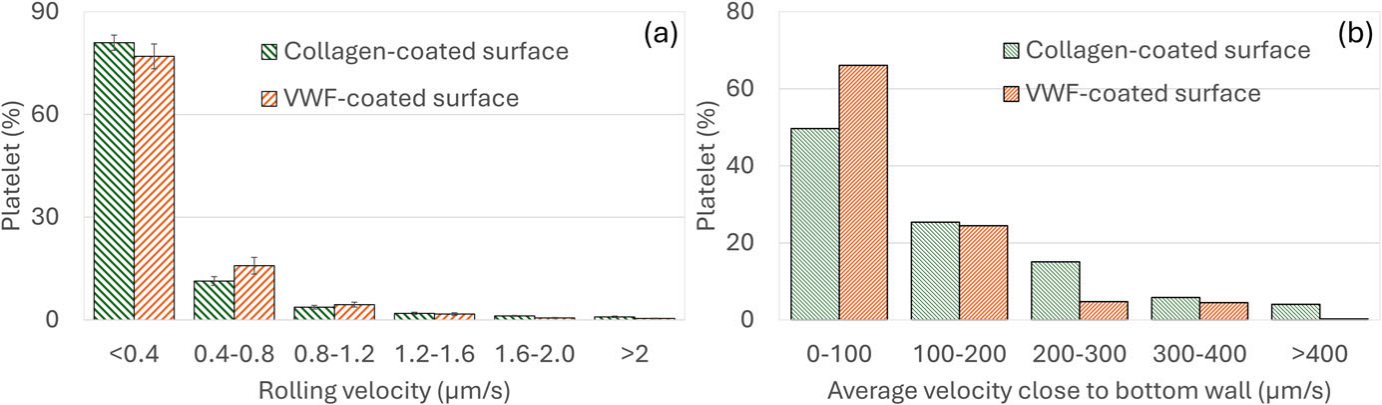
**a** Percentage of platelets within six platelet rolling velocity categories over collagen- and VWF-coated surfaces from the microfluidic experiment. **b** Percentage of platelets within $1l_{DPD}$ from the coated surface under six average velocity categories from DPD simulations

Figure 8a shows the histogram of platelets under various rolling velocity categories to indicate transient adhesive interaction with the coated surfaces, and platelet slowing down before adhesion. Platelet slowing down indicates transient adhesive receptor-ligand binding to initiate platelet adhesion. The rolling velocity is plotted as the average of the eight experimental observations, for both the collagen- and VWF- coated surfaces, after 1 min of perfusion. Figure 8a indicates that more than 75% of the platelets close to the channel wall have a rolling velocity below $0.4 \mu m/s$ for both collagen- and VWF- coated surfaces, indicating temporary adhesion before firm adhesion. The percentage of platelets falling under various rolling velocity categories is similar for collagen- and VWF coated surfaces, indicating similar initial transient temporary adhesion phase. In our DPD simulations, we did not explicitly model the platelet rolling mechanism, thus we only show the platelet average velocities within $1l_{DPD}$ wall distance from the coated surfaces in Fig. 8b. Figure 8b shows the percentage of platelets, close to the bottom wall, within various ranges of average velocity categories from the DPD simulation. More than 50% of the platelets have velocities less than $100 \mu m/s$ which is very low compared to the plasma particle average velocity ~ 4 mm/s (see supplements), indicating that platelets are slowing down due to stochastic bond formation and breaking. The platelet percentage with < 100 $\mu m/s$ velocity near wall is slightly higher for VWF-coated surfaces, which is expected as platelet-VWF bonds form and break more rapidly compared to platelet-collagen coated surface. Note, the values are multiple orders of magnitude higher than the rolling velocities observed in experiments, because the platelets, due to the stochastic nature of our modeling, are simultaneously attached to and detached from the adhered surface, rather than rolling continuously on the surface. Fig. S3 in the supplement shows the variation in platelet velocity close to channel wall for different values of characteristic bond force $F_{0}$.

**Fig. 9:**
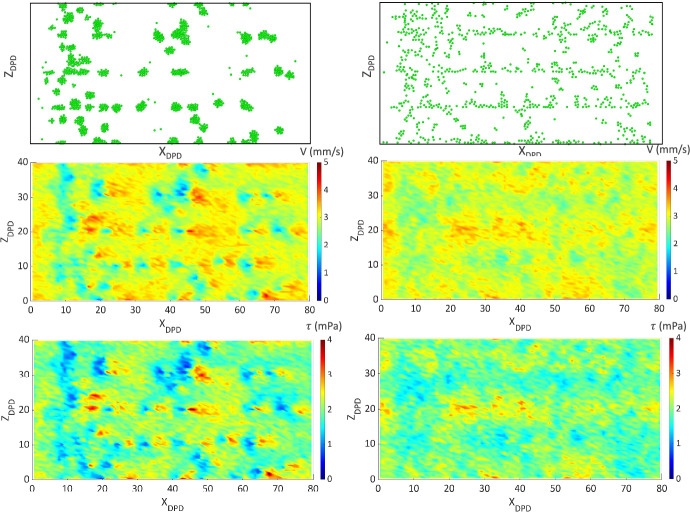
Variations in plasma velocity ($V_{DPD}$) and shear stress ($\tau_{DPD}$) due to adhered platelets over collagen (left)- and VWF (right)-coated surfaces

**Fig. 10:**
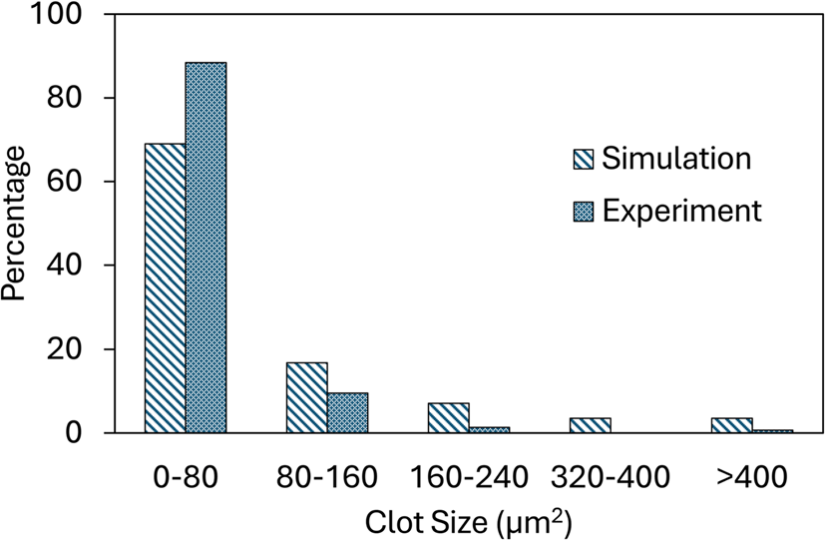
Platelet aggregate size distribution over collagen-coated surface

Figure 9 shows contours of plasma velocity (V), and shear stress ($\tau$) near the collagen- (left) and VWF- (right)coated surfaces. As platelets adhere and begin to aggregate, plasma velocity decreases locally within the forming clot region, whereas the velocity increases in the surrounding area to maintain a constant flow rate. Thus, variations in local velocity and shear stress are observed: increased shear stress at the region of high velocity and decreased shear stress at the region of low velocity. These spatial variations in shear and velocity may promote the scattered, multi-layered clots on collagen-coated surfaces. In particular, the higher fluid forces surrounding a forming thrombus increase the probability of bond detachment at the clot periphery, preventing spreading of platelets across the surface even though collagen is fully coated on the substrate. On the contrary, variations in plasma velocity and shear stress over the VWF-coated surfaces are less compared to the collagen-coated surface. Due to less variation in plasma velocity and shear stress, the platelet-covered area reaches a nearly a plateau value after some time of perfusion. Figure 10 shows the platelet aggregate size distribution after 5 min of perfusion over a collagen-coated surface for both simulation and experimental observations. (The aggregate size distribution over the VWF-coated area is not shown, since platelets continuously translocate throughout the domain; no stable clot is formed.) The simulated size distribution shows a good match with the experimental observation, where ~ 80% thrombi have a size below 80 $\mu m^{2}$, and ~ 3% are above 400 $\mu m^{2}$.

## Discussion

Platelet adhesion at the vascular injury site is a very complex multistep process. Initially, platelets attach to the exposed subendothelial matrix via the interactions of GPIbα with VWF, and GPVI and α2β1 with collagen, which initiates platelet morphological change, secretion of agonists (e.g. Adenosine diphosphate, ADP), platelet activation (GPIIb/IIIa conformational change), leading to platelet firm adhesion on the binding site (Barnes et al. 1998; Yip et al. 2005). The later stage involves GPIIb/IIIa mediated binding with other platelets to form firm fibrin-enhanced platelet aggregation (Barnes et al. 1998; Yip et al. 2005). It was found that GPIbα-VWF, GPVI-collagen and α2β1-collagen bindings are essential for platelet adhesion, whereas GPVI-collagen bonds primarily initiate platelet activation (GPIIb/IIIa receptor activation) (Nuyttens et al. 2011), which is mandatory for permanent clot formation on the surface (Moroi et al. 1996).

Here we present a phenomenological model for platelet adhesion and aggregation dynamics on the collagen- and VWF-coated surfaces to capture based on experimental observations. Our goal is to adapt a simple probabilistic approach that can predict the platelet adhesion dynamics over collagen- and VWF-coated surfaces observed during in vitro experiments. We adopted a coarse-grained simulation approach, dissipative particle dynamics (DPD), to simulate blood flow through collagen- and VWF-coated microvascular channels. To reduce the complexity of the system, we do not explicitly introduce VWF unfolding in the model, but rather represent platelet adhesion and aggregation dynamics through a viscoelastic attractive/dissipative force model. The multistage process of clot formation on the coated surface was achieved by introducing two distinct stages, $t_{1}$ and $t_{2}$ with different model constants during platelet adhesion, where the first stage depicts a weaker GPIb $\alpha$-VWF or GPVI-collagen bond and the final state depicts a stronger, fibrin-enhanced clot through GPIIb/IIIa binding (Wang et al. 2020). The scattered clot formation on the coated surface was achieved by the introduction of a probability function that depends on the external fluid flow. This probabilistic approach prevents incoming platelets from covering the whole area of the coated surface. Note that, as the complete bottom surface of the computational domain is covered with either collagen or VWF particles, the incoming platelets, near the coated surface and within the cutoff radius$r_{c}$, have the affinity to create a bond with the coated-surface particles. Thus, after a long time, in the limit of $t \to \infty ,$ without the stochastic approach, the entire coated surface will be covered with platelets. The introduction of the stochastic modeling allows the incoming platelets to adhere to and simultaneously detach from selected places based on$P_{dis}$. .

The predicted platelet adhesion and aggregation on the collagen-coated surface with our DPD simulation model matched the experimental clot formation pattern both qualitatively and quantitatively. We plotted simulated platelet-covered area variation with time along with experimental results, showing that the simulated data matched the experimental observations. In the DPD model, $a_{0}$ is the elastic parameter of the viscoelastic adhesion model governing the attractive force and determines the binding strength of two intersecting particles at each timestep. A larger $a_{0}$ would increase the covered area percentage on the coated surface. We choose to report only the model parameters that closely resemble the experimental data. With the increase of time, the platelet-covered area increased monotonically, indicating scattered multi-layered clots on the collagen-coated surface. In both our DPD and experimental observations, we found scattered multilayer clots covering the collagen-coated surface. Due to the activation of platelets, adhered platelets recruit additional platelets to form multilayer clots. Our DPD-mulated results capture this physics through $P_{dis}$, which depends on the local shear force ($F/F_{0}$). In the narrow region, between the already adhered platelets, high local shear increases the probability of detachment, and platelets deposit on top of one another to form multilayer clots. It should be noted that, if the clots get bigger, vertical to the flow direction, at some point in time, flow-induced drag might rupture a portion of the big clot, which flows with the flowing plasma in the vascular system to create embolism (Wang et al. 2020; Tosenberger et al. 2012). Due to the stochastic process, at each timestep, new platelets are recruited and some platelets are detached from the top of the clot surface.

Platelets bind on the VWF-coated surface with a relatively weaker GPIbα-VWF bond which rapidly breaks, and as a result, we observe scattered single isolated platelet adhesion over the VWF-coated surface (Fig. 7). This phenomenon was achieved by the increasing damping coefficient $\gamma_{p}$ of the viscoelastic model. Although the elastic coefficient $a_{0}$ (Eq. 9) remains the same, as used for collagen simulations, the increase in damping effect (Eq. 10) reduces relative motion between an already adhered platelet and an incoming platelet. As a result, rather than attaching on top of the adhered platelet, incoming platelet slides pass it and attaches over a nearby VWF-coated surface. This might have helped to reduce clustering (Fig. 5) and form a single isolated-type platelet adhesion (Fig. 7). As the VWF-coated surface gets saturated with single platelet layers, after some time (> 100s), the growth rate of the platelet-covered area slowed down while the total number of platelets adhered on the vWF surface leveled. Our simulated results are consistent with the experimental observations both, qualitatively and quantitatively.

In our model, the saturation of platelet coverage over VWF-coated surfaces emerges from the combined effects of the force-dependent detachment probability $P_{dis} \left( F \right)$​ and progressive occupation of available local binding sites on the coated surface. As platelet adhesion proceeds, increasing surface occupancy reduces the number of accessible attachment sites for incoming platelets. At the same time, the relatively weak and rapidly breaking platelet-VWF bonds, represented through elevated damping, promote frequent detachment events. This dynamic balance between attachment and detachment leads to a steady-state coverage level. $P_{dis} \left( F \right)$ governs the stability of newly formed bonds, while surface occupancy limits further recruitment, resulting in a saturation regime when attachment and detachment rates become comparable. The flow redistribution and reduced shear variation observed in Fig. 9 therefore arise as a consequence of this stabilized adhesion pattern.

Platelet rolling represents a transient adhesive interaction with the coated surface, during which the platelets experience reduced velocity close to the surface. This slowing down happens due to the temporary initial receptor–ligand engagement to initiate the adhesion process, increasing the likelihood of firm adhesion. The distribution of platelets under various rolling velocity categories, therefore, indicates the dynamic balance between hydrodynamic shear force and adhesive interaction. A higher platelet percentage in the lower velocity categories suggests stronger adhesive interactions, platelet activation, and the possibility of stable adhesion later. Conversely, higher rolling velocities indicate weaker interactions and a lower probability of subsequent activation and adhesion. We have observed similar platelet percentages for different rolling velocity categories for the collagen- and VWF-coated surfaces.

Although our DPD model predictions match the experimental results closely, there are certain limitations in our implementation. We only focused on investigating the clot formation dynamics (single and multilayer) on the collagen- and VWF-coated surfaces. We did not take into account the effect of individual receptor contribution in clot formation, rather considered that, platelet-platelet and platelet-coated-surface interactions happen via a spring-dashpot viscoelastic model system within a cut-off radius. During hemostasis and thrombotic events, red blood cells (RBCs), white blood cells, agonists (ADP/ATP), platelets, and plasma VWF all contribute together (Li et al. 2025), however, we only consider the interaction between platelet, coated surface and plasma particles to simplify the model. Future investigations can include incorporating VWF unfolding and fragmentation dynamics into the model to provide physiological insights into shear-driven platelet activation and adhesion.

We acknowledge that the absence of a strict no-slip condition represents a limitation of the present DPD approach, and wall slip may introduce some quantitative deviation in the absolute magnitude of near-wall forces. We calibrated the global shear rate to closely match experimental conditions (~ 500 s^−1^), ensuring that the overall hydrodynamic loading on adhered platelets remains physiologically relevant. Moreover, the force-dependent detachment model parameters ($k_{0}$ and $F_{0}$) were systematically adjusted to reproduce experimentally observed platelet-covered area and adhesion dynamics for both collagen- and VWF-coated surfaces. The good quantitative agreement between simulations and in vitro measurements (Figs. 4 and 6) suggests that any bias introduced by wall slip is compensated within the calibrated parameter space and does not lead to systematic over- or under-estimation of adhesion stability in our study. Our present work focuses on comparative platelet aggregation trends between collagen- and VWF-coated surfaces under identical hydrodynamic conditions. Since the same near-wall flow characteristics and modeling assumptions apply to both cases, relative differences in adhesion patterns and detachment dynamics remain robust. Future work incorporating improved wall models may further refine near-wall force resolution and provide more direct correspondence with physiological shear environments.

## Conclusion

We have implemented a coarse-grained dissipative particle dynamics (DPD) model and performed simulations along with experiments with human blood to understand the platelet adhesion and aggregation dynamics on the collagen- and VWF-coated surfaces. A model based on viscoelastic adhesion-dissipation, along with a probabilistic platelet attachment/detachment function, was implemented. Two distinct types of platelet adhesion dynamics on the collagen- and VWF-coated surfaces were quantitatively and qualitatively captured. The collagen-coated surface shows multilayers of scattered deposited platelets, while the VWF-coated surface shows scattered single-isolated-type platelet adhesion. Platelets’ affinities towards collagen and VWF are very distinct, where platelets bond with collagen via GPVI and later form firm aggregation with the help of GPIIb/IIIa induced fibrin network with other platelets, and platelets usually form weaker bonds with VWF through its GPIbα, which rapidly breaks down and acts as a mediator between the platelet and the exposed subendothelial surface. Future investigations are necessary to understand how already damaged platelets form clots over an injured site, as receptor shedding alters platelet activation and adhesion.

## Supplementary Information

Below is the link to the electronic supplementary material.

Supplementary file1 (PDF 44 KB)

Supplementary file2 (PDF 154 KB)

Supplementary file3 (PDF 1121 KB)

## Data Availability

The data supporting the findings of this study are available from the corresponding author upon reasonable request.
