# Supplementary figures and images for: Platelet Adhesion and Aggregation Dynamics over Collagen- and VWF-coated Surfaces: Insights from Dissipative Particle Dynamics Simulations and Microfluidic Experiments
Source: Bull Math Biol. 2026 Mar 4;88(4):50. doi: 10.1007/s11538-026-01615-5 (PMC12960359; doi:10.1007/s11538-026-01615-5)

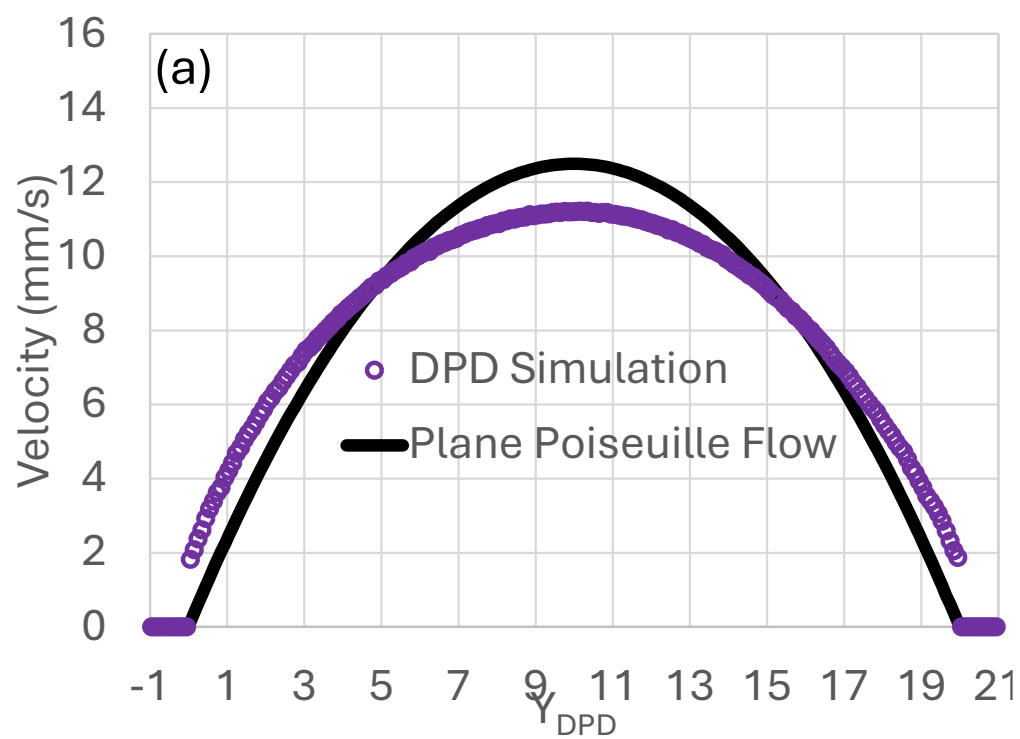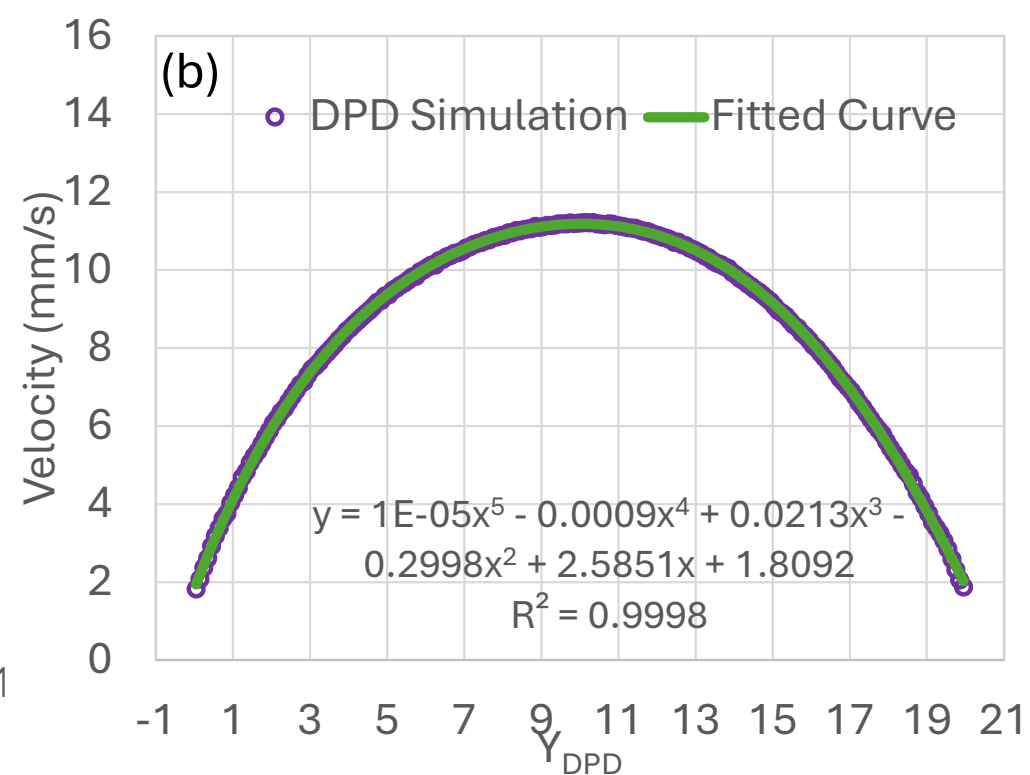

Figure S1: Plasma velocity profile in the channel

Supplement: Supplementary file 1 — Supplementary file1 (PDF 44 KB) [file 11538_2026_1615_MOESM1_ESM.pdf]

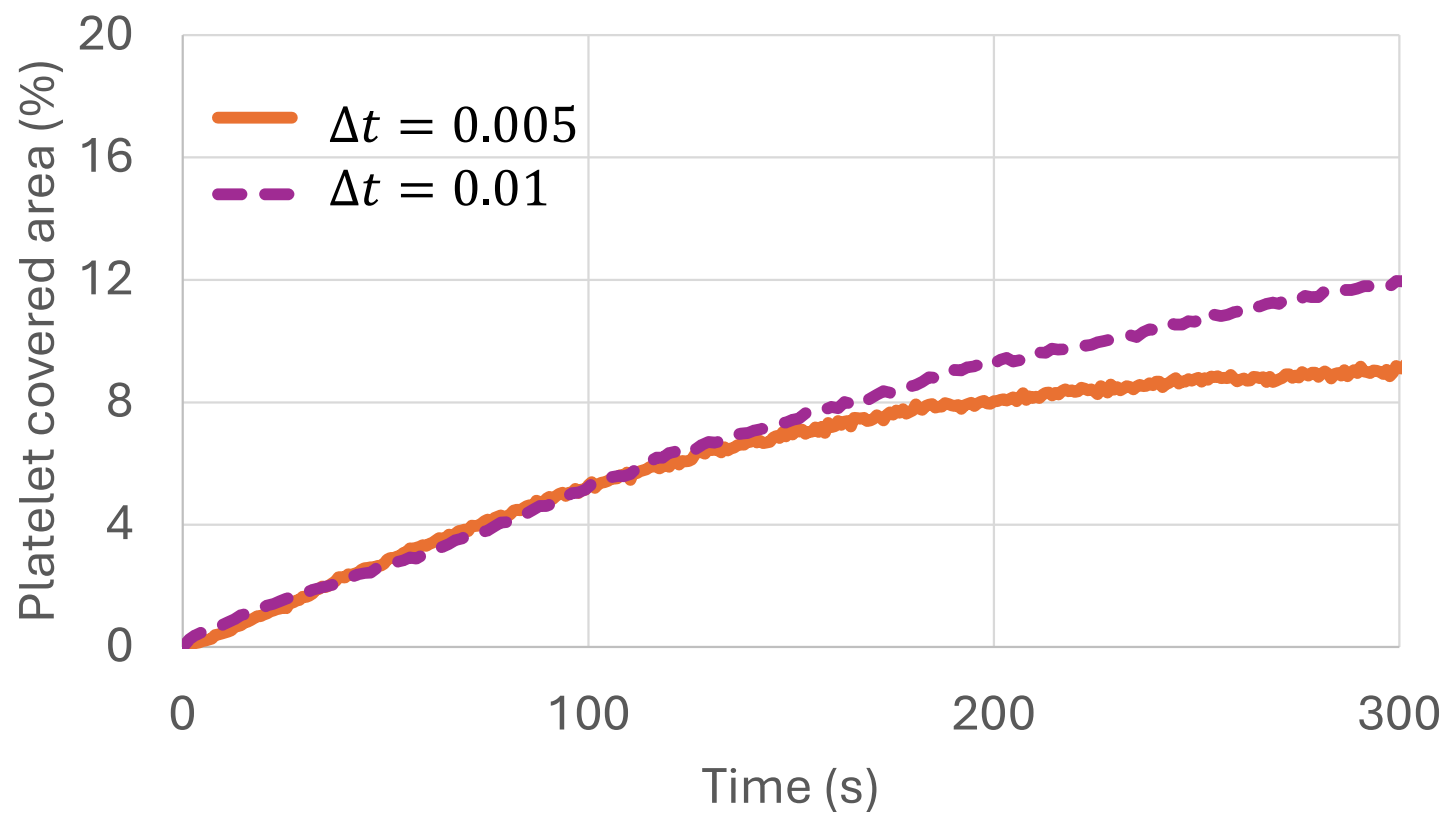

Figure S2: Variation in platelet covered area for two different timesteps.

Supplement: Supplementary file 2 — Supplementary file2 (PDF 154 KB) [file 11538_2026_1615_MOESM2_ESM.pdf]
