# Supplementary material for: Platelet Adhesion and Aggregation Dynamics over Collagen- and VWF-coated Surfaces: Insights from Dissipative Particle Dynamics Simulations and Microfluidic Experiments
Source: Bull Math Biol. 2026 Mar 4;88(4):50. doi: 10.1007/s11538-026-01615-5 (PMC12960359; doi:10.1007/s11538-026-01615-5)
Supplement: Supplementary file 3 — Supplementary file3 (PDF 1121 KB) [file 11538_2026_1615_MOESM3_ESM.pdf]

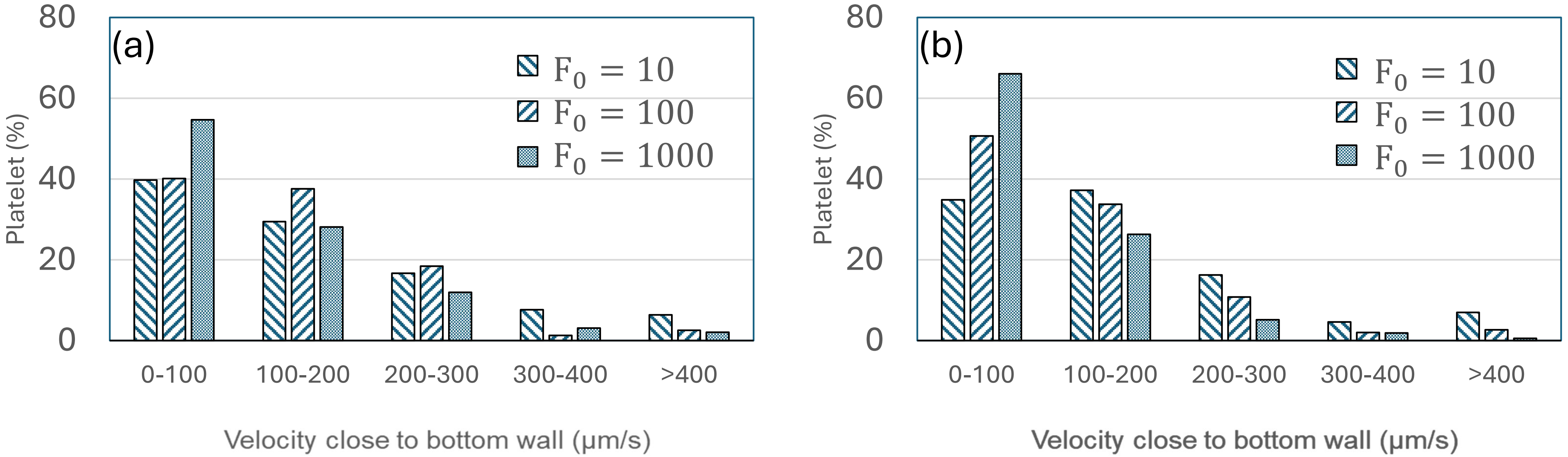

Figure S3: Percentage of platelets within $1l_{DPD}$ from the (a) collagen-coated surface and (b) VWF-coated surface for various characteristic bond force F_0 under six average velocity categories from DPD simulations
